# Age at menarche and the menstrual pattern of secondary school adolescents in northwest Ethiopia

**DOI:** 10.1186/1472-6874-9-29

**Published:** 2009-10-05

**Authors:** Desalegn Tegabu Zegeye, Berihun Megabiaw, Abay Mulu

**Affiliations:** 1 Department of Epidemiology and Biostatistics, University of Gondar, PO Box-196, Gondar, Ethiopia; 2 Department of Human Anatomy, University of Gondar, PO Box-196, Gondar, Ethiopia

## Abstract

**Background:**

Population studies on normal and dysfunctional characteristics of menstrual cycles are scarce in Ethiopia. In addition variability in menarcheal age and menstrual characteristics are common. Knowledge on this variability is necessary for patient education and to guide clinical evaluation.

**Methods:**

A cross sectional study was conducted in two small towns called Dabat and Kola Diba, northwest Ethiopia between April and May 2007. Systematic sampling method was used to select 622 school girls from two secondary schools. A pretested questionnaire prepared in Amharic was used to gather data. Selected girls cooperated in answering the questionnaire in their classrooms under the supervision of the research team. Only 612 of the adolescent females were included in the final analysis, of which 305 were from Koladiba High School and 307 from Dabat.

**Results:**

The age of the study subjects ranges between 14 and 19 with a mean (standard deviation) of 16.9 ± 1 years. About 92.2% had attained menarche by the time the survey was conducted. The probit analysis of the *status quo *data yielded a median (CI) age at menarche of 14.8 (13.9-15.3) years. The average age at menarche by recall method was 15.8 ± 1 years. The mean age at menarche was 0.3 years younger for urban females compared with rural ones (p < 0.001). A cycle length between 21 and 35 days was observed in 70.3% of the girls. The mean duration of flow was 4 ± 1.3 days with a range of 2-7 days. The menstrual cycles were irregular in 42.8% of the subjects. The overall prevalence of dysmenorrhoea was 72% among these subjects. Premenstrual symptoms were present in 435 of the females (75.4%). The leading sources of menarcheal information to the adolescents were mothers (39.7%), followed by their friends (26.6%) and teachers (21.8%).

**Conclusion:**

In this study age of menarche was found to be delayed which is even higher than the findings indicated similar studies conducted in Ethiopia and other African countries. A significant number of students complain of abnormal menstrual cycle, dysmenorrhoea and premenstrual symptoms which call for appropriate counselling and management.

## Background

Menarche is the onset of menstruation and it is one of the most significant milestones in a woman's life. The mean age at menarche varies from population to population and is known to be a sensitive indicator of various characteristics of population including nutritional status, geographical location, environmental conditions and magnitude of socioeconomic inequalities in a society [1-3]. Studies suggested that menarche tends to appear earlier in life as the sanitary, nutritional and economic conditions of a society improve [4,5]. For most females, it occurs between the age of 10 and 16 years; however, it shows a remarkable range of variation[3]. The normal range for ovulatory cycles is between 21 and 35 days. While most periods last from three to five days, duration of menstrual flow normally ranges from two to seven days. For the first few years after menarche, irregular and longer cycles are common [3,6,7].

Knowledge of the length and variation of the menstrual cycle is necessary for patient education and for identifying deviations from normal to guide clinical evaluation[7]. Among the gynaecological problems, menstrual problems are said to be the major ones especially among adolescent females [6,7]. These disorders are often the source of anxiety for female adolescents and their families at large. The common menstrual disorders for female adolescents are amenorrhea, abnormal/excessive uterine bleeding, dysmenorrhea, and premenstrual syndrome [6,7].

Population studies on normal and dysfunctional characteristics of menstrual cycles are scarce in Ethiopia. The purpose of this paper is therefore, to determine the age at menarche and patterns of menstruation among secondary school girls, and to identify the magnitude of common menstrual disorders.

## Methods

### Study design

A cross sectional study was conducted in two small towns of Dabat and Kola Diba, northwest Ethiopia, between April and May 2007. These districts were selected for the reason that they are University of Gondar Team Training Program field sites. The study sites have also presented another comparative advantage, each representing a highland and a lowland area respectively.

### Study area

Dabat, a highland area with an altitude of 2754 meters above sea level, is found at a distance of 70 kms to the North of the city of Gondar. It has an estimated population of 15,870[8]. On the other hand, Kola Diba, a lowland area 2000 meters below sea level has a population of 16,035. It is located at a distance of 35 kms to the South of the city of Gondar [9].

### Sampling

There was one high school in each of the study areas. Only grade 9 and 10 students were selected for the survey because the majority of 9^th ^and 10^th ^graders fall in the ideal age groups to study post menarcheal menstruation pattern. Sample size was calculated assuming 95% confidence level, 50% high risk prevalence (for the sake of having larger sample size it was considered taking 50% as appropriate) and acceptable difference of 4%.

Assuming a non response rate of 10% the total sample size stood at 622.

Equal number of students from the schools of Koladiba and Dabat were enrolled. In each classroom a systematic sampling method was used to identify the sample population after the male students made leave the classrooms. All female students (622) cooperated in answering the questionnaire in their classrooms under the supervision of the research team. Only 612 of the female students were included in the final analysis, partly because many of the girls did not report their exact dates of birth or age at menarche. Of the 612 girls, 305 were from Koladiba while 307 were from Dabat High School.

### Data collection

A self-administered questionnaire prepared in Amharic, the mother tongue of all the respondents, and pre-tested on school girls of a similar nearby area was used for collecting the most relevant data from the study subjects. The questionnaire included socio demographic information about the respondent's age, marital status, education level, ethnic group and residence. Localities with 2000 or more inhabitants are considered urban.

Questions related to menstruation comprised menarcheal age, menstrual pattern (regularity of cycle, cycle length, duration of flow, amount of flow), premenstrual symptoms, dysmenorrhea and severity of dysmenorrhea and associated symptoms, impact of menstrual disorder on school attendance, the source of information about menarche and whether they required medical help (from a physician, nurse or midwife) for menstrual disorder or not.

The age at menarche was determined by questioning the girl if she had or not had her menstruation and at which age she had menstruated (0.5 years were systematically added to the age obtained). Then the ages of menarche were grouped in three classes of menarche (early, medium and late). Early menarche is considered 12 years or less, medium menarche between 13 and 14 years and delayed menarche more than 14 years[10,11].

Explanation on the relevance of the study to the female students was given before they filled the questionnaire. Besides, male students were not in the class while females were filling the questionnaires. Questionnaires were distributed and collected on the same day to ensure confidentiality and prevent information contamination.

### Data analysis

The data were analyzed using Epi Info 2000(CDC, Atlanta, Georgia) and SPSS v13 (SPSS Inc, Chicago). Data were analyzed using the χ^2 ^test, t- test and logistic regression analysis. For information obtained by status quo procedure the age at menarche was calculated by probit analysis. *P *< 0.05 was considered to show statistical significance.

However, owing to some missing answers to certain survey questions, the denominator used in percentage computation varies according to the responses obtained for the survey question.

Ethical approval was obtained from the Institutional Review Board of the College of Medicine and Health Sciences of the University of Gondar. Permission to conduct the study was also obtained from the principal's of participating schools. The purpose of the study was explained to the students and verbal consent was obtained from them regarding their agreement to participate in the study. No name was recorded in order to keep the identity of respondents anonymous.

## Results

From 622 distributed questionnaires 612 were filled correctly (98.4%). Half (50.2%) of the respondents were from Dabat. Amharas constitute 99.7% of the population and 92.8% were Orthodox Christians. About 61% were from rural areas. Their age ranges between 14.5 and 19.5 years with a mean and standard deviation of 17.4 ± 1 years. Only 44(7.2%) were married (Table 1).

**Table 1:** Socio-demographic characteristics of female school adolescents in northwest Ethiopia, March 2007.

**Variable**	**no (%)**
**Age**	
14.5	2(0.3)
15.5	36(5.9)
16.5	181(29.6)
17.5	194(31.7)
18.5	177(28.9)
19.5	22(3.6)

**District**	
Dabat	307(50.2)
Koladiba	305(49.8)

**Residence**	
Urban	237(38.7)
Rural	375(61.3)

**Grade**	
9^th^	406(66.3)
10^th^	206(33.7)

**Religion**	
Orthodox	568(92.8)
Muslim	33(5.4)
Other	11(1.8)

**Ethnicity**	
Amhara	610(99.6)
Other	2(0.4)

**Marital status**	
Single	560(91.5)
Married	44(7.1)
Divorced	8(1.4)

Forty seven (7.7%) of the subjects did not have their first menstruation yet at the time of interview (Table 2). Their age range between 14.5-18.5 and mean age was 16.4 ± 0.8 years. These groups of students were not included in the analysis of recall age at menarche, menstrual pattern and disorder.

**Table 2:** Age distribution of menstruating girls in northwest Ethiopia

**Age**	**Menses started**		**Total**
	**Yes**	**No**	

14.5	1	1	2

15.5	24	12	36

16.5	154	27	181

17.5	190	4	194

18.5	174	3	177

19.5	22	0	22

**Total**	565	47	612

By using probit analysis, the median age at menarche was found to 14.8 (13.9-15.3) years. The data used for probit analysis revealed that the distribution did not differ from the normal (Pearson X^2 ^= 3.93, d.f. = 4, p = 0.415). The age at menarche by recall method was 15.8 ± 1.0 years with a range of 11.5 to18.5 years (Table 3). Nine female adolescents (1.6%) have an early menarche (≤ 12.5 years), 87 girls (15.4%) had a medium menarche (13 and 14 years) and 469(83%) experienced a delayed menarche (≥ 15 years). The subjects living in rural areas revealed a mean and median age at menarche of 15.9 and 15.1 years respectively. The mean age at menarche was 0.3 years younger for urban girls compared to rural girls which is statistically significant with p value < 0.001(Table 3).

**Table 3:** Mean and median age at menarche, northwest Ethiopia.

**Variable**	**Probit analysis**		**Recall age**			
	**n**	**median (95% CI)**	**n**	**mean ± sd**	**t**	**P- value**

District						
Dabat	286	15.3(14.5-15.8)	294	15.9 ± 1	2.2	>0.06
Koladiba	279	14.4(12.2-15.2)	284	15.7 ± 1		

Residence						
Rural	343	15.1(10.3-16)	349	15.9 ± 1	3.26	<0.001
Urban	222	14.4(11.9-15.2)	229	15.6 ± 1		

**Total**	**565**	14.8(13.9-15.3)*	**578**	**15.8 ± 1**		

*X^2 ^= 3.92, df = 4, p = 0.415

There were no statistically significant differences (t = 2.2, p-value > 0.06) between mean menarcheal ages of Koladiba (15.7 ± 1) and that of Dabat (15.9 ± 1) female students who took part in the study (Table 3).

The menstrual pattern of study subjects is shown in table 4 with respect to district, place of residence and age of menarche. The menstrual cycles were irregular in 42.8% of the study subjects. The proportion of girls with irregular cycle was higher in Koladiba (47.3%) as compared to Dabat (38.4%) (P value < 0.01). There was no statistically significant difference of menstrual cycle regularity by residence (urban vs rural) or age of menarche.

**Table 4:** Menstrual pattern of Secondary school adolescents in northwest Ethiopia, March 2007.

**Menstrual characteristics**	**Total (%)**	**District**		**Residence**		**Gynaecologic age**		
		**Dabat (n = 286)**	**Koladiba (n = 279)**	**Rural (n = 343)**	**Urban (n = 222)**	**<2 years (n = 432)**	**3-4 years (n = 121)**	**>5 years (n = 12)**

**Cycle length**								
<14 days	49 (8.6)	27	22	23	26	34	15	0
14-20	68 (12)	46	22	42	26	51	14	3
21-28	322(57)	148	173	191	130	248	66	7
29-35	76(13.4)	39	37	48	28	53	21	2
>35 days	51 (9)	28	26	39	12	46	5	0

**Duration**								
2-3 days	262(46.7)	134	128	163	99	207	49	6
4-5 days	229(40.8)	114	115	140	89	165	60	4
6-7 days	70(12.5)	38	32	38	32	56	12	2

**Regularity**								
Regular	323(57.2)	176	147	188	135	252	66	5
Irregular	242(42.8)	110	132	155	87	180	55	7

Out of the total study population, 117(20.7%) adolescents had a menstrual cycle length shorter than 21 days, 51(9%) longer than 35 days and 70% had a cycle length between 21 and 35 days. There is a statistically significant difference on cycle length among the subjects living in Dabat and Koladiba district (X^2 ^= 10.9, p-value < 0.03). The difference between the length of the cycle by place of residence (urban, rural) is also significant (X^2 ^= 9.6, p-value < 0.05).

The duration of menstruation was taken to the nearest whole day, as information about the hour at which menstruation began and ceased could not be obtained. The mean duration of flow was 4 ± 1.3 days with a range of 2-7 days. The difference between these mean values was not statistically significant by district, residence or age of menarche.

Only 37.6% use sanitary pad and in these group the average number of pads used per day was 3 ± 1.84 (range 1-12). The rest 353(62.4%) use pieces of clothes as sanitary goggle and it was impossible to determine the size and number of pieces used. The proportion of girls who used sanitary pads was higher in urban areas (57.9%) as compared to rural girls (42.1%) (P value < 0.01). Average number of pads used per day was also found more in urban girls (3.4 ± 2 pads per day) than rural girls (2.7 ± 1.5) which is statistically significant with p-value < 0.001.

The overall prevalence of dysmenorrhoea (assumed to be primary dysmenorrhoea, as secondary is rare at this age) was 72%, and among these subjects of the study, it was rated mild in 295 (73%), moderate in 58(14.4%) and sever type in 54 (12.6%). Dysmenorrhoea was significantly more frequent among students from a rural residence, in those from Koladiba, those who said they had an irregular cycle, those with longer duration of bleeding (>6 days) and those with long cycles (> 28 days) (See table 5). However, dysmenorrhoea was not significantly associated with age at menarche. Dysmenorrhoea resulted in absence from school of 196(48%) girls with dysmenorrhoea. Among girls who had dysmenorrhoea, only 46(11.4%) had consulted a health worker about the problem.

**Table 5:** Dysmenorrhoea and Premenstrual syndrome among School adolescents in northwest Ethiopia, March 2007.

**Variable**	**Dysmenorrhoea**			**PMS**		
	**Yes (%)**	**X^2^**	**P value**	**Yes (%)**	**X^2^**	**P value**

District		6.9	**<0.004**		11.8	**<0.001**
Koladiba	215(77.1)			228(81.7)		
Dabat	192(67)			198(69.2)		

Residence		7.1	**<0.004**		0.7	0.19
Rural	261(76)			263(76.7)		
Urban	146(65.8)			163(73.4)		

Onset of menarche		0.52	0.76		3.2	0.2
Early	18(78.3)			20(87)		
Medium	219(71.3)			236(76.9)		
Late	170(72.2)			170(72.3)		

Cycle length		10.39	**0.03**		5.2	0.26
<21 days	71(60.6)			81(69.2)		
21-28	238(74.1)			245(76.3)		
29-35	59(77.6)			59(77.6)		
>35	39(76.5)			41(80.4)		

Duration		32.2	**<0.001**		9.3	0.09
2-3	163(62.2)			186(71.4)		
4-5	181(79)			177(77.2)		
6-7	61(85.7)			59(82)		

Regularity		16.8	**<0.001**		19.7	**<0.001**
Regular	211(65.3)			221(68.4)		
Irregular	196(81)			205(84.7)		

The results of the study (Table 5) indicated that there was a significant association between the severity of dysmenorrhoea and the duration of menstrual flow (P < 0.02). High level of severe dysmenorrhea was observed among adolescents with irregular cycle (60.8%) as compared to those with regular cycle (39.2%) with p < 0.03). More female adolescents with moderate to severe dysmenorrhoea (68%), as compared to mild (43.4%) were absent from school because of the pain (p < 0.001). Severity of dysmenorrhoea was unaffected by the length of menstrual cycle, gynecologic age, age of menarche, chronological age or visit of health professional for the pain(Table 6).

**Table 6:** Factors associated with severity of dysmenorrhoea among school adolescents in northwest Ethiopia, March 2007.

	**Dysmenorrhoea**			**X^2 ^(df)**	**P value**
	**Mild (%)**	**Moderate (%)**	**Severe (%)**		
	**n = 299**	**n = 62**	**n = 54**		

**Cycle length**					
<21 days	48(16.2)	12(20.7)	10(18.6)	6.68(6)	0.57
22-28 days	177(60)	30(51.7)	30(58.8)		
29-35 days	41(13.9)	12(20.7)	5(9.8)		
>35 days	29(9.8)	4(6.9)	6(11.8)		

**Menses duration**					
2-3 days	133(44.9)	15(26.3)	16(32)	22.3(4)	**<0.02**
4-5 days	121(41.1)	35(61.4)	23(46)		
6-8 days	41(13.9)	7(12.3)	11(22)		

**Regularity**					
Irregular	130(44.1)	33(56.9)	31(60.8)	7(2)	**<0.03**
regular	165(55.9)	25(43.1)	20(39.2)		

**Absent from school**					
Yes	117(39.9)	43(74.1)	36(70.6)	33.8(2)	**<0.001**
No	176(60.1)	15(25.9)	15(29.4)		

**Visited Health professional**					
Yes	30(10.2)	10(17.2)	5(9.8)	2.5(2)	0.28
No	263(89.8)	48(82.1)	46(90.2)		

Premenstrual symptoms were present in 426 girls (75.4%). The most common symptom was abdominal cramp, which was experienced by 330 (76.2%) of those with PMS. There was statistically significant association between presence of premenstrual symptoms and district of residence and regularity of the cycle (Table 4). PMS was not significantly associated with age at menarche, duration of menses, cycle length (Table 4). A significantly higher proportion of female adolescents who had premenstrual symptoms also suffered from dysmenorrhoea (82.4%) compared to adolescents who had no premenstrual symptoms (40.3%).

From the total study subjects, 564 (92.1%) reported they had got information about menarche from someone. The leading sources of menarcheal information were mothers (39.7%), followed by friends (26.6%) and teachers (21.8%).

## Discussion

Menarche is an important event in a woman's life showing (directly or indirectly) many socioeconomic, environmental, nutritional and geographical differences in societies [4,12-14]. Age of menarche was found to be delayed by both probit analysis (14.8 year) and recall method (15.8 year) in this study which is even higher than similar study in the same region by Haile and Roth two decades ago[15]. Even higher than a study done more than three decades ago in Addis Ababa, Ethiopia, which was 13.6 ± 1.5 years [16]. Another study conducted in Addis Ababa reported a lesser mean age of menarche as 13.72 ± 1.31 years[17]. The probit analysis also showed that the menarcheal age in our study was also found higher than many African countries [Nigeria 13.19 ± 1.32 years for urban and 14.22 years for rural areas[18], Sudan = 13.85[19], Morocco = 13.66[11], Mozambique 13.9 ± 1.29[20]. As age and age at menarche were asked as age in whole years, the data about age and age at menarche may not be very precise.

There was no significant difference in the mean age at menarche of the two areas of different altitudes in our study i.e. Dabat (15.9 ± 1) versus Koladiba (15.7 ± 1) p > 0.06 which disagree with a study in Peru[21] and India [22] which showed an increase in the mean age of menarche with altitude. The reason may be the small sample size of the study or Dabat is not situated at high enough attitude to significantly affect the age at menarche.

The mean age of menarche for rural female adolescents was significantly higher than for urban ones, which is in agreement with the result found in Nigeria[18] and Morocco[11]. This can be explained by the better socioeconomic status for urban girls than rural ones and the lack of fat for rural girls due to malnutrition. Besides, rural girls travel long distances to school every day which may partially put them to stress and delay their menarche.

About 30% of the study subjects had experienced abnormal menstrual cycle length (<21 days or >35 days) which is a common phenomenon in the first two years after menarche[6]. This is because anovolation is common at this time [6,7]. Other causes of menstrual irregularity like endocrine disorders, tumours and acquired disorders like stress, strenuous exercise and cigarette smoking are potential topics for further research.

In this study dysmenorrhea (pain during menstruation) was reported by 72% of the study subjects of which about 28.5% were having moderate to severe dysmenorrhea. A study done among Malaysian school girls [23] showed a similar result (67.7%) with ours. In our study, dysmenorrhea was more common among those who had irregular cycles, longer duration of flow, rural residents, from Koladiba district and those who had premenstrual syndrome. In the study done in the US by Banikarim et al, dysmenorrhea was the leading cause of short-term school absenteeism [24] which is similar to our study in which about 48.8% of those suffering from dysmenorrhea had reported to be absent from school merely due to the pain. This again agrees well with many studies which reported a 34-50% of school absenteeism due to dysmenorrhea [23,25-27].

About 75.4% percent of adolescent female students had at least one symptom of PMS in the last 6 month. It is similar to a study done in Malaysia (75%) [23] but lower than the figure reported in a study done among Jimma University students[28] where 99.6% of students had at least one premenstrual symptom in the last 12 months. Since PMS is a leading cause of school absenteeism and unnecessary suffering, it is important that girls are given counselling and offered proper guidance.

In this study 67.4% of the girls reported that they use homemade sanitary pads which is slightly higher than the finding shown in a study done in Addis Ababa(47.8) [17]. Attempts to measure menstrual blood loss on the basis of number of pads or tampons used per day or frequency of pad changes was not successful because of variations in individual fastidiousness, type and brands of sanitation product used and individual estimation of volume of flow.

Menarche may remain a traumatic event for a female adolescent unless she was prepared for it[29]. Mothers were found as potential sources of information in urban girls where as teachers for rural girls. This agrees with studies done in India [30] and Malaysia [23]. The difference in the source of information between rural and urban girls may be due to poor knowledge of rural mothers about menstruation which makes them refrain from advising their daughters about menstruation and related reproductive health issues.

## Conclusion

Compared to similar studies carried out in other African countries, menarcheal age was found to be delayed in our study. Age at menarche varies by residence i.e. later among rural subjects of the study than urban ones. Dysmenorrhea was a common problem in school adolescents with significant short-term school absenteeism however, there still exists less practice of medical consultation for the problem though there is high prevalence of dysmenorrhea. Mothers and teachers were potential sources of information on menarche for school girls in urban and rural girls respectively.

## Competing interests

The authors declare that they have no competing interests.

## Authors' contributions

DTZ wrote the proposal, participated in data collection, analyzed the data and drafted the paper. BM and AM approved the proposal with some revisions, participated in data collection and analysis. DTZ, BM and AM revised subsequent drafts of the paper. All authors read and approved the final manuscript.

## Pre-publication history

The pre-publication history for this paper can be accessed here:


